# Evaluation of primary stability of innovated orthodontic miniscrew system (STS): An *ex-vivo* study

**DOI:** 10.4317/jced.52676

**Published:** 2016-07-01

**Authors:** Massoud Seifi, Negin-Sadat Matini

**Affiliations:** 1 DDS, MSD, MS Med Edu. Dentofacial Deformities Research Center, Shahid Beheshti University of Medical Sciences, Tehran, Iran; 2 DDS. Department of Orthodontics, Shahid Beheshti University of Medical Sciences, Tehran, Iran

## Abstract

**Background:**

Stability is determined as one of the requirements in use of Temporary Anchorage Devices (TAD) in orthodontics. Miniscrew has been a widely used Bone Anchor. Compared with mini-implant that necessitates osseointegration; mechanical retention is a determining factor for primary stability of miniscrew. Studies investigated various ways to increase primary stability. The aim of this study is to introduce a new configuration of miniscrew system which is believed to obtain more primary stability.

**Material and Methods:**

Freshly ovine mandibles were cut in blocks. Twenty-seven miniscrews (diameter 1.6 × 8 mm; G2, Dual Top Anchor System, Jeil Medical, Seoul, Korea) were inserted in the blocks and divided in 2 experimental groups: single miniscrew and the innovated design “Seifi Twin Screw (STS)”. Primary stability was evaluated by Periotest “M”® device.

**Results:**

Independent t-test showed a significant difference between 2 experimental groups in periotest evaluation (*p*< 0.05). STS demonstrated higher primary stability due to its mechanical configuration and design.

**Conclusions:**

The STS provides higher primary stability and was found to be effective in increased success rate of miniscrew systems from the standpoint of primary stability.

** Key words:**Anchorage procedures, anchorage techniques, orthodontic anchorage procedures, miniscrews, temporary anchorage device.

## Introduction

Stable Anchorage is one of the major factor in successful orthodontic treatment. Skeletal anchorage is used as one of the temporary anchorage devices (TAD), especially in complicated cases. Miniscrews are examples for skeletal anchorage which are used widely in different sites of mandible and maxilla (1,2). They reduce the need for dental anchorage and can provide different tooth movements without patient’s cooperation. There are other advantages of miniscrews as TADs such as non-invasive insertion procedure, providing rigid anchorage against orthodontic loads and minimal anatomic limitation for placement (2,3). However, there are still problems which have effects on the success rate of miniscrew-assisted treatments. Because of immediate loading on orthodontic miniscrews, primary stability became a basic requirement for loading forces on miniscrews (4,5). It is considered as clinical condition of miniscrew immobility and capacity to withstand loads in different directions (6). The primary stability of miniscrews is mostly supported by mechanical retention between bone and miniscrew surface (7,8). Primary stability is influenced by factors such as overloading (5), bone density (6,9-11), cortical bone thickness (12), screw design (13,14) and root proximity (15).

Studies about different variety of miniscrew designs to improve primary stability are increasing. Different changes in screw diameter, length and the design of the threads have been investigated (16).

There are different methods to assess miniscrew primary stability. Measuring insertion torque, resonance frequency analysis (RFA) and periotest value (PTV). The force used to insert the implant is called insertion torque (17), insertion torque is related to bone tissue, cortical bone thickness and bone density. Adequate insertion torque is an indicator of mini implant stability (14). It should be as high to ensure stability and as low enough to prevent overcompression of the bone. Resonance frequency analysis is also another method for quantitative measurement of primary stability, RFA value is assessed by attaching a transducer directly to the implant (18). In this device, a magnetic piece called “SmartPeg” is screwed on top of the implant head. A handpiece emits electromagnetic impulses to SmartPeg in order to detect the resonance frequency of SmartPeg implant unit (4). A noninvasive device called periotest is used for analysis of implant stability. This device originally developed to measure damping effect of periodontal ligament around natural tooth. The range of PTV depends on damping characteristic of periodontal ligament around tooth (13). It can also assess the mobility of implants and it has been used to measure primary stability of orthodontic mini-screws. The periotest device (Medzintechnik Gulden, Modautal, Germany), produces a transient vibration by tapping the implant as a rod inside the periotest handpiece which is electromagnetically accelerated. The device shows a quantitative reading from -8 (clinically rigid) to +50 (very mobile) (19). More negative PTV means more stability of the implant. Wireless Periotest device (Periotest “M”) is the recent design introduced for measuring stability in implant and orthodontic miniscrew. It is easy to use in clinical approach and shows reasonable and reproducible results from implant-bone interface (19).

All methods above have been evaluated in different studies. RFA and PTV are noninvasive measurements for stability and they have shown reliability and sensitivity (18).

We designed an innovated system, Seifi Twin Screws (STS), for skeletal anchorage by coupled miniscrews. The system has been used for force eruption and extrusion of impacted canine by the author. The author suggests that this system demonstrates more primary stability compared with previous designs.

The aim of our study is to introduce the innovated STS and evaluate the primary stability of it compared with conventional single miniscrew anchorage system by PTV measurements.

## Material and Methods

-Specimen Preparation

Freshly ovine mandibles were cut into 10 cm long pieces under profuse saline-solution cooling (legal permission was obtained from Institutional Review Board). A total of 18 bone blocks were prepared after removing soft tissue. To determine cortical bone thickness and trabecular bone density, each bone block was scanned by Cone Beam Computed Tomography (CBCT) unit (NewTomVGi, Verona, Italy). The radiation exposure was set on High Resolution with 6cm × 6cm Field Of View (FOV). In order to equalize specimens, areas of similar cortical bone thickness and trabeculation were assessed as the insertion site of miniscrews for each bone block. The assessment of the scans was performed by the manufacturer recommended software (NNT Viewer). Then, bone blocks were allocated in two groups of single miniscrew and the innovated system (STS), each group contained 9 blocks.

-Experimental Groups and Configuration of STS

**Figure 1:**
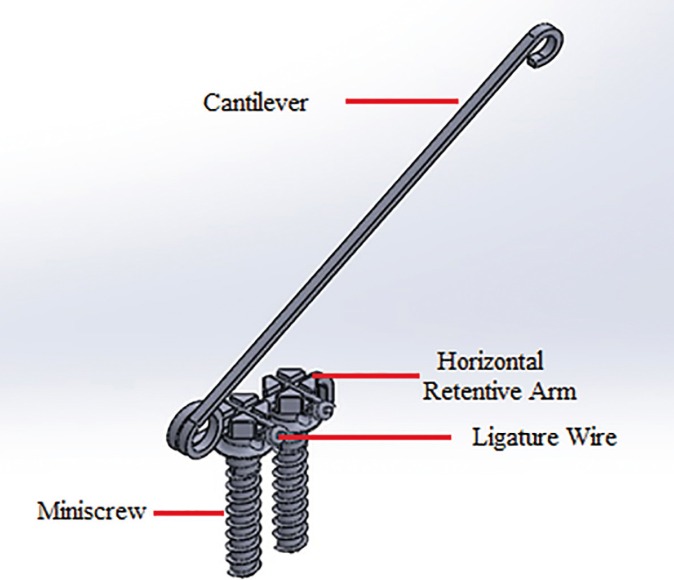
Seifi Twin Screw (STS): Schematic view.

Twenty-seven self-drilling orthodontic miniscrews (diameter 1.6 × 8 mm; G2, Dual Top Anchor System, Jeil Medical, Seoul, Korea) were used. The insertion of all miniscrews were done by a single operator. For single miniscrew group, one miniscrew was inserted perpendicular to the bone surface of each block assisted by hand-held screw driver (Dual Top Anchor System, Jeil Medical, Seoul, Korea) according to the manufacturer. All miniscrews were placed with no pilot hole. For group two (STS), one mini-screw was first inserted to block; then, the second miniscrew was inserted parallel to the first one with the distance that the tissue suppression stops of both miniscrews were in contact at the proximal aspect. To complete the configuration of the STS, an 18” × 25” stainless steel rectangular wire (Dentaurum, Ispringen, Deutschland), passed through both miniscrews slots; this wire act as horizontal retentive arm and were fixed by ligature wires (Dentaurum, Ispringen, Deutschland) which were engaged in eyelets that were placed at the neck of the miniscrews (Schematic configuration of STS is illustrated in figure 1).

-Primary Stability measurements

**Figure 2:**
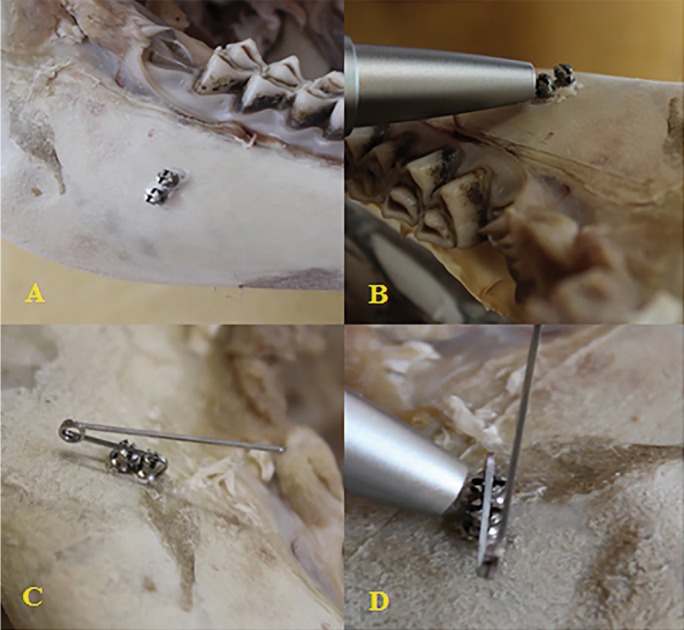
A) Insertion of both miniscrews in bone block. B) Periotest evaluation on a single screw. C) STS configuration is completed and the wire is in place. D) Evaluation of periotest for STS.

The primary stability measurement was conducted using the periotest “M”® device (Medzintechnik Gulden, Modautal, Germany). According to the manufacturer, the tip of the periotest was placed perpendicular to the miniscrew and was held approximately 2mm away from the miniscrew head. This device measures the time that the rod remains in contact with the miniscrew; shorter contact time indicates more stability of miniscrew. Values were detected three times for each sample and entered to Excel 2013 for further analysis. Because of the contact between tissue platforms and the wire, the STS was considered as a single unit; so, based on the pilot study which showed no significant difference in PTV between two miniscrews in STS; the periotest measurements were only performed for one of the miniscrews for each system (Fig. 2).

-Statistical Analysis

Data were tested for normal distribution by Kolmogorov-Smirnov test. The independent t-test was performed for comparison of PTV between two experimental groups using statistical software SPSS (Statistical Package for the Social Sciences, New York, USA) version 21.

## Results

The mean (SD) values of PTV in single screw and STS groups are displayed in  Table 1. The Kolmogorov-Smirnov test showed normal distribution for the PTV values in both experimental groups (*p*=0.2). The independent t-test revealed significant difference between single screw and STS groups for PTV. The mean value of PTV in the innovated system was significantly higher than the single screw system (*p*=0.025).

**Table 1:**
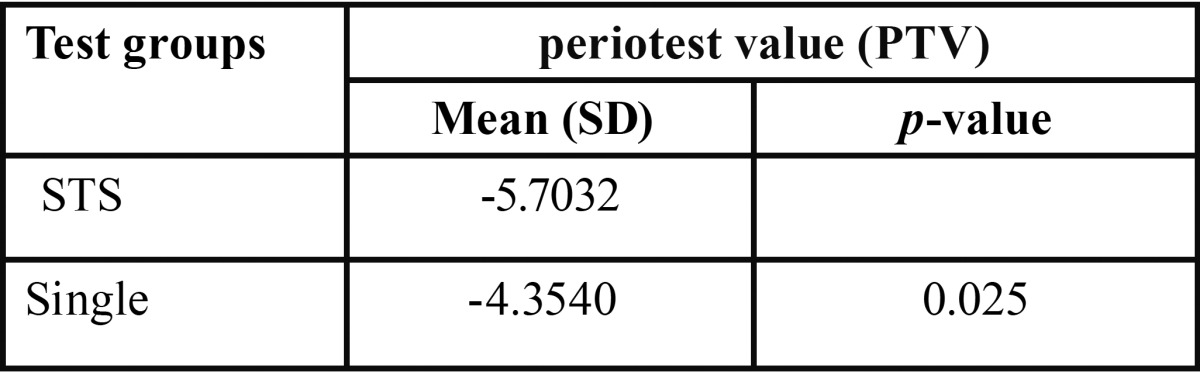
Evaluation of PTV in test groups.

**Figure 3:**
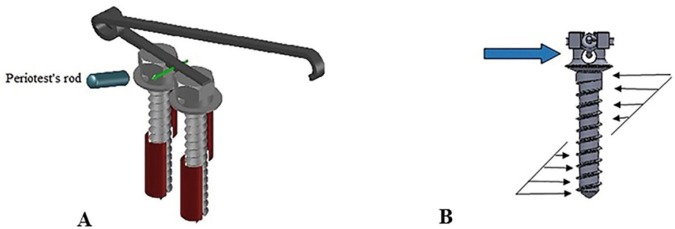
A) Periotest Evaluation on STS. B) Force distribution by applying periotest’s rod on a single miniscrew.

Based on results regarding increased stability in STS. A force distribution can be analyzed and compared between single screw system and STS. As demonstrated in figure 3. In STS, as we applied periotest’s rod perpendicular to the long axis of miniscrew; the horizontal retentive arm between screws transfers the force to the other miniscrew and it resists against displacement and caries out a part of the force. The maximum tension probably is decreased and increased stability results reduced micro movements of miniscrews due to periotest evaluation.

In the other hand, when we applied force to the single miniscrew system, statically, the force is resisted by a triangular distribution around the center of rotation. In application of single miniscrew, maximized reaction is produced in top and bottom of the mini-screw body, which produces excessive tension to the surrounded bone and reduced stability.

## Discussion

Stability has a notable effect in achieving successful skeletal anchorage. Studies demonstrated different factors that have correlation with stability of orthodontic miniscrews (20,21). Because of increasing the need for immediate loading, primary stability is the utmost importance (22). The stability should be checked immediately after the insertion of miniscrew, any evidence of mini-screw loosening within the bone results failure of orthodontic treatment in nearly future (20). Primary stability depends on the mechanical engagement of miniscrew and bone; hence, it does not require a period for osseointegration (17). Lack of primary stability can lead to mobility of the miniscrew and subsequently failure of the treatment (23). Bone quality and quantity at the receptor site (11,12), design of the miniscrew (10) and insertion technique (24) are the factors to be considered to ensure primary stability. Due to the lack of clinicians control over bone quality and quantity, miniscrew design and insertion techniques can be the variables in order to enhance success of the procedure. Self-drilling miniscrews doesn’t require predrilling of the bone prior the miniscrew insertion. This technique improves the mechanical interlock between bone and the device. Several studies reported wider and longer miniscrews in spite of increase bone-screw interface and contact area (25). A study by Nienkemper *et al.* (26) described higher stability of 11 mm mini-implants in initial insertion at midpalatal region compared with 9 mm mini-implants. In contrast to the mentioned study (26), 8 mm miniscrews were used for STS and it showed increased primary stability in consistent with previous retrospective studies (27).

The area of miniscrew insertion is also concerned. Many investigators believe that the maxilla has more success rate for minis-crews compared to mandible. The palatal area is superior option for miniscrews insertion and the first author include midpalatal area for the insertion of STS.

Without any change to the shape of miniscrews, we have introduced a novel designed system for skeletal anchorage i.e. two orthodontic self-drilling miniscrews (diameter 1.6×8mm). The advantages of self-drilling miniscrews compared with self-tapping are less operation time, less bone debris, decreasing thermal damage and also patient’s comfort (5). Both miniscrews were connected by a rectangular wire which passes through both miniscrew slots and acts as a horizontal retentive arm. The wire was locked in the slots by ligature wires that were tied through built-in eyelets on the neck of each miniscrew. Based on our results, this system (STS), improves primary stability in comparison to the single miniscrew anchorage. The above mentioned design i.e. STS has increased contact surface with the bone. The horizontal retentive arm that has linked both miniscrews to each other creates a truss. The “truss effect” organized two components; makes both miniscrews behave as a single object. The Truss creates shearing force between two miniscrews in eccentricity. It resist distortion from applied forces in any direction and fastened the system in all degrees of freedom. Authors believe that STS is more stable than single miniscrew because it can resists counteracting moments and forces in X, Y, and Z axes.

Periotest “M”® device is a reliable indicator for measurement of implants stability in both conventional and immediate loadings. This device has a high capacity regarding the determination of miniscrew stability/looseness in *in-vitro* studies. The PTV quantitative results from STS showed more stability compared with single miniscrew (*p*<0.05). In other words, STS is able to reduce the amount of time which the rod of the periotest is in contact with the miniscrew (the technology which is used for quantitative assay).

The present study represents an innovated design for skeletal anchorage devices without any intervention to the miniscrew designed by the manufacturer. Tozlu *et al.* (28), has created an apparatus (a miniscrew ring) which was placed at the neck of the screw. The mentioned study claimed that this ring is able to increase stability due to increasing surface contact of bone with miniscrew. It also has spines which resist from the loading forces; punching the tissue is required to insert spines. Our study is based on the effect of a truss support to prevent moments and forces which cause rotation of miniscrews in bone and eventually minis-crew mobility after the application of load. STS does not require tissue punch or any additional manufactured apparatus and the configuration can be done by conventional orthodontic appliances (miniscrew, rectangular wire, ligature wire).

Youn *et al.* (7), demonstrated the stability of Hollow type miniscrew compared with C-type miniscrew in beagle dogs. The newly designed H-type miniscrew has 4 fenestration holes for ingrowth of bone. Results concluded that the use of H-type depends on local bone quality and it can be used in maxilla and the C-type in mandible. Also, the fenestration holes in H-type miniscrew is designed to increase osseointegration and it does not have an effect on primary stability. STS is made by miniscrews that can be inserted in most areas of maxilla and mandible. This system has increased surface contact of bone at the insertion site which improves mechanical interlocking of bone and miniscrew.

In clinical approach, anatomical consideration is another limitation for miniscrew insertion. Authors suggest midpalatal area for the insertion of STS which has reduced risk of root damage and has enough bone structure for insertion of miniscrews as can be evaluated in coronal sections of CBCT’s. Studies claimed that tongue irritation may occurred when miniscrew is placed in palatal area. Applying bonding resin and composites (flow-able or regular body) on the miniscrew heads in STS creates a smooth surface and diminish plaque accumulation.

Histologic evaluation and tissue response to the miniscrews have a bulk of literature support (17,24) but the present study was conducted on the ovine bone blocks for studying the mechanics of the forces and moments applied and the counteracting elements of the system. Clinical cases will determine the feasibility of this system in practice and adequate data regarding the efficacy of the STS will be published in near future in conjunction with the available data. Further studies are needed to investigate stability overtime by applying different types of force on STS.

## Conclusions

The innovated system of STS, has showed increased primary stability compared to a single miniscrew. The STS configuration can distribute the impacted energy in a larger area and in various orientations for counteracting unwanted dislodging forces or moments. The quantitative evaluations suggest that STS can be used as an advantageous skeletal anchorage device in orthodontic treatments.
